# Induced Ferromagnetism in Epitaxial Uranium Dioxide Thin Films

**DOI:** 10.1002/advs.202203473

**Published:** 2022-10-09

**Authors:** Yogesh Sharma, Binod Paudel, Amanda Huon, Matthew M. Schneider, Pinku Roy, Zachary Corey, Rico Schönemann, Andrew C. Jones, Marcelo Jaime, Dmitry A. Yarotski, Timothy Charlton, Michael R. Fitzsimmons, Quanxi Jia, Michael T. Pettes, Ping Yang, Aiping Chen

**Affiliations:** ^1^ Center for Integrated Nanotechnologies (CINT) Los Alamos National Laboratory Los Alamos NM 87545 USA; ^2^ Glenn T. Seaborg Institute Los Alamos National Laboratory Los Alamos NM 87545 USA; ^3^ Neutron Scattering Division Oak Ridge National Laboratory Oak Ridge TN 37831 USA; ^4^ Materials Science and Technology Division Oak Ridge National Laboratory Oak Ridge TN 37831 USA; ^5^ Materials Science and Technology Division Los Alamos National Laboratory Los Alamos NM 87545 USA; ^6^ Department of Materials Design and Innovation University at Buffalo The State University of New York Buffalo NY 14260 USA; ^7^ National High Magnetic Field Laboratory (NHMFL) Los Alamos National Laboratory Los Alamos NM 87545 USA; ^8^ Department of Physics and Astronomy University of Tennessee Knoxville TN 37996 USA; ^9^ Present address: Department of Physics Saint Joseph's University Philadelphia PA 19131 USA

**Keywords:** actinide materials, epitaxy, lattice‐strain, magnetism, thin films, uranium dioxide

## Abstract

Actinide materials have various applications that range from nuclear energy to quantum computing. Most current efforts have focused on bulk actinide materials. Tuning functional properties by using strain engineering in epitaxial thin films is largely lacking. Using uranium dioxide (UO_2_) as a model system, in this work, the authors explore strain engineering in actinide epitaxial thin films and investigate the origin of induced ferromagnetism in an antiferromagnet UO_2_. It is found that UO_2+_ *_x_* thin films are hypostoichiometric (*x*<0) with in‐plane tensile strain, while they are hyperstoichiometric (*x*>0) with in‐plane compressive strain. Different from strain engineering in non‐actinide oxide thin films, the epitaxial strain in UO_2_ is accommodated by point defects such as vacancies and interstitials due to the low formation energy. Both epitaxial strain and strain relaxation induced point defects such as oxygen/uranium vacancies and oxygen/uranium interstitials can distort magnetic structure and result in magnetic moments. This work reveals the correlation among strain, point defects and ferromagnetism in strain engineered UO_2+_ *_x_* thin films and the results offer new opportunities to understand the influence of coupled order parameters on the emergent properties of many other actinide thin films.

## Graphical abstract

This work uses epitaxial strain to tune the oxygen stoichiometry and magnetism in UO_2+_ *_x_* thin films. The UO_2+_ *_x_* films are hypostoichiometric (*x*<0) when in‐plane lattice is tensile strained, while films are hyperstoichiometric (*x*>0) when in‐plane lattice is compressively strained. Induced ferromagnetism is attributed to the epitaxial strain and strain relaxation‐induced point defects such as oxygen vacancy and oxygen interstitials.

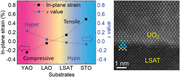

## 1 Introduction

Actinide materials are an important part of the modern technological world as they play crucial roles in various applications that range from nuclear energy to quantum computing.^[^ ^1^, ^2^, ^3^ ^]^ For example, UO_2_ is the main form of nuclear fuel used in the current generation of nuclear reactors.^[^ ^1^, ^4^ ^]^ In addition, UTe_2_ that hosts Majorana fermions could be used for topological quantum computation.^[^ ^5^, ^6^ ^]^ Unfortunately, the state‐of‐the‐art efforts on actinide materials have been mainly limited to bulks. The famous phrase that “the interface is the device” referred to the great success of devices based on semiconductor films for electronic/photonic applications that started over half a century ago.^[^ ^7^ ^]^ During the past decades, we have witnessed tremendous breakthroughs in complex oxides enabled by thin‐film growth technology. Strain engineering, interface engineering, and defect engineering have been widely used to tune functional properties or create exotic phenomena in thin films. There is a strong need to apply these engineering methods to actinide materials towards their applications. By using the actinide thin film capability established at Los Alamos National Laboratory, we report strain engineering of epitaxial actinide thin films, specifically, UO_2_. The reasons to study UO_2_ are the following: It is of fundamental interest as members of the class of strongly correlated materials.^[^ ^8^, ^9^, ^10^, ^11^ ^]^ Theoretical studies propose the effect of electron correlation and order parameter coupling that can lead to unique functionalities in UO_2_.^[^ ^8^, ^9^, ^11^, ^12^, ^13^, ^14^ ^]^ Exploring spin‐lattice coupling may generate insights into the thermophysical properties, which are key factors to optimize the performance of UO_2_ as a nuclear fuel material.^[^ ^8^, ^15^ ^]^ Despite extensive theoretical research efforts over the past few decades, experimental studies on physical behaviors that emerge through strongly coupled structural, electronic, and magnetic degrees of freedom are few in UO_2_.^[^ ^16^, ^17^, ^18^, ^19^, ^20^ ^]^

UO_2_ has the face‐centered cubic (fcc) fluorite (CaF_2_) structure with lattice parameter *a* = 5.471 Å at 300 K, where the uranium (U^4+^) cations reside on an fcc lattice with eight nearest‐neighbor oxygen (O^2−^) anions forming a cube.^[^ ^21^ ^]^ In bulk UO_2_, neutron‐scattering measurements revealed a non‐collinear spin ordering with the transverse triple‐q (3‐k) structure.^[^ ^22^, ^23^, ^24^ ^]^ Below a first‐order phase transition at *T*_N_ = 30.8 K, the magnetic dipole moment adopts long‐range AFM order accompanied by a Jahn–Teller distortion of the oxygen sublattice.^[^ ^17^ ^]^ Recent studies show the existence of a piezomagnetic effect^[^ ^16^ ^]^ and hydrostatic pressure‐induced ferromagnetic ordering below *T*_N_ in bulk UO_2_.^[^ ^25^ ^]^ Observations of piezomagnetism can be seen in terms of a robust magneto‐elastic memory effect in bulk single crystal UO_2_.^[^ ^16^ ^]^ Pressure‐induced weak ferromagnetism can be observed in powdered UO_2_.^[^ ^25^ ^]^ The strong spin‐lattice coupling can be observed in terms of changing the net magnetic moment through a change in UO_2_ lattice dimension.^[^ ^16^ ^]^ The spin‐lattice coupling is of fundamental interest to understand the low thermal conductivity of UO_2_.^[^ ^15^, ^18^ ^]^ These early results on bulk UO_2_ open paths to manipulate the physical properties of UO_2_ using external stimuli, such as mechanical strain.

Strain engineering in epitaxial thin films has been applied to a variety of non‐actinide thin films in the past decade.^[^ ^26^, ^27^, ^28^, ^29^, ^30^ ^]^ A lattice mismatch of a few percent between a film and the underlying substrate can produce several GPa of biaxial pressure.^[^ ^31^, ^32^ ^]^ This epitaxial strain can be used to manipulate the electronic and magnetic ground states in actinide thin films. Reports in some other oxide thin films have demonstrated a variety of strain relaxation mechanisms, including misfit dislocations, microstructure modulation, cation stoichiometry modulation, and oxygen vacancy modulation.^[^ ^33^, ^34^, ^35^ ^]^ Therefore, both epitaxial strain and strain‐relaxation induced effects can affect functional properties. Here, we report the epitaxial growth of UO_2_ thin films and explore the strain engineering in epitaxial UO_2_ thin films. We found out that UO_2+_ *_x_* thin films, strained on STO substrate (in‐plane tensile strain), are hypostoichiometric with *x*<0. UO_2+_ *_x_* thin films grown on LAO and YAO substrates with in‐plane compressive strain are hyperstoichiometric with *x*>0. The incorporation of point defects is a critical way to accommodate epitaxial strain in UO_2+_ *_x_* thin films. Epitaxial strain and strain relaxation induced point defects such as oxygen/uranium vacancies and oxygen/uranium interstitials could distort the noncollinear magnetic structure and result in magnetic moments in UO_2+_ *_x_* thin films.

## 2 Results and Discussion

**Figure 1:**
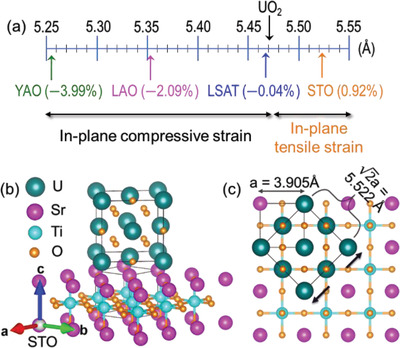
a) Lattice mismatch between bulk UO_2_ and various perovskite substrates. b) Crystal structure schematic of (001) UO_2_ epitaxially grown on (001) STO, depicting the 45° rotation of the UO_2_ unit cell (above) with respect to the cubic STO lattice (below). c) Epitaxial orientation relationship between the surface mesh of the (001) UO_2_ film and underlying (001) STO substrate, with one UO_2_ unit cell highlighted, which corresponds to 0.92% lattice mismatch.

We selected 4 substrates with different lattice parameters to potentially tune the epitaxial strain states of UO_2_ films. They are YAlO_3_(YAO, $\sqrt{2}$a ≈ 5.260 Å), LaAlO_3_(LAO,$\;\sqrt{2}$a ≈ 5.358 Å), (La,Sr)(Al,Ta)O_3_ (LSAT, $\sqrt{2}$a ≈ 5.468 Å) and SrTiO_3_ (STO, $\sqrt{2}$a ≈ 5.521 Å). Bulk UO_2_ has a lattice parameter *a_bulk_* = 5.471 Å.^[^ ^4^ ^]^ **Figure** **1**a illustrates the in‐plane lattice mismatch between bulk UO_2_ and these substrates. The epitaxial strain and lattice parameters are shown in **Table** **1**. Epitaxial growth occurs due to the crystallographic alignment between the perovskite‐oxide substrate and the cubic fluorite structure of UO_2_ with a 45^o^ in‐plane rotation. By taking an example of UO_2_ growth on STO substrate, the epitaxial orientation relationship between the UO_2_ lattice and substrate as well as the surface mesh of (001) UO_2_ film and underlying (001) substrate are shown in Figures 1b,c.

**Table 1:** Summary of observed lattice parameters, in‐plane lattice mismatch between substrate and bulk UO_2_, calculated in‐plane strain, Poisson ratio and unit cell volume from RSMs

Substrate	Substrate lattice in diagonal [Å]	Lattice mismatch *f* [***%***]	***a***_∥_ of film [Å]	***a***_⊥_ of film [Å]	In‐plane strain [%]	Out‐of‐plane lattice c [%]	Poisson ratio *ν*	Unit cell volume [Å^3^]
UO_2_ bulk	5.471						0.33	163.75
UO_2_/YAO	5.260	−3.857	5.458	5.429	−0.237	‐0.767	–	161.73
UO_2_/LAO	5.538	−2.065	5.469	5.463	−0.037	‐0.146	–	163.39
UO_2_/LSAT	5.468	−0.055	5.479	5.468	0.146	‐0.055	–	164.14
UO_2_/STO	5.521	0.914	5.498	5.455	0.493	−0.292	0.23	164.89

**Figure 2:**
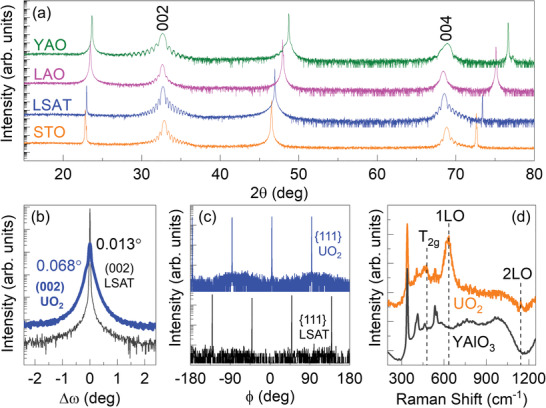
a) *θ*–2*θ* scans of single‐phase (001)‐oriented UO_2_ films on different substrates. b) Rocking curves of the (002) peaks for LSAT substrate and UO_2_ film. c) *ϕ*‐scans of LSAT {111} and UO_2_ {111} family planes. d) Raman spectra of the UO_2_/YAO heterostructure and the spectrum from a reference YAO substrate, which show the presence of all phonon modes akin to the fluorite structure of UO_2_.

**Figure** **2**a shows XRD longitudinal scans for UO_2_ thin films. Only 00*l* reflections of the UO_2_ films are observed in the *θ–*2*θ* scans, indicating that all the films are 00l textured and phase pure. Clear thickness fringes are observed around the main diffraction peaks, confirming a uniform thickness and smooth interface for all films. The UO_2_ film thickness estimated using these fringes in the diffraction pattern is around 21, 22, 19, and 17 nm on STO, LSAT, LAO, and YAO substrates, respectively. To further confirm the crystalline quality of the films, rocking curves and azimuthal *ϕ*‐scans are measured. For example, rocking curve scans around the (002) reflection of the UO_2_ film grown on LSAT substrate are shown in Figure 2b. The full width at half maximum (FWHM) of the (002) rocking curve is measured to be ≈0.068° and ≈0.013° for the UO_2_ layer and the underlying LSAT substrate, respectively, which indicates the high‐quality epitaxial UO_2_ film. As determined from the X‐ray *ϕ*‐scans in Figure 2c, the growth of UO_2_ shows a cube‐on‐cube epitaxy with a 45^o^ in‐plane rotation. The in‐plane epitaxial orientation relationship between the film and substrate is (001) UO_2_ || (001) LSAT and [110] UO_2_ || [010] LSAT.

Figure  2d shows the room‐temperature deep ultraviolet Raman spectrum of the UO_2_ film grown on YAO and the Raman spectrum of the YAO bare substrate. Based on the symmetry assignment, the Raman spectrum of UO_2_ film confirms the presence of three phonon modes: first and second‐order longitudinal optical (LO) phonon modes near 630 and 1145 cm^−1^ and a T_2g_ mode located near 470 cm^−1^. These modes correspond to the fluorite structural symmetry of bulk UO_2_.^[^ ^36^ ^]^ Note that the oxidation states of epitaxial UO_2_ films have been confirmed using X‐ray photoelectron spectroscopy.^[^ ^37^ ^]^

**Figure 3:**
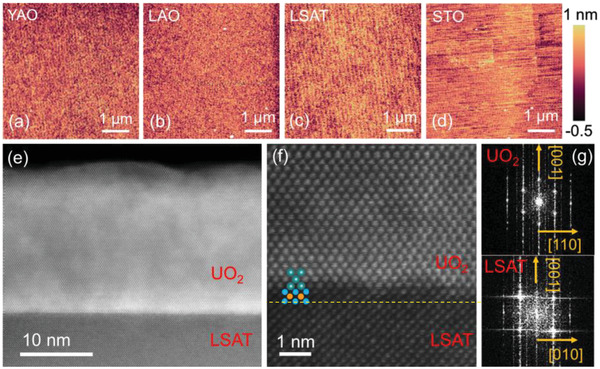
a–d) Atomic force microscopy images showing atomically smooth surfaces with step terrace structures for the films grown on YAO, LAO, LSAT, and STO substrates. e) A cross‐sectional STEM image taken along the [001] LSAT zone axis. f) STEM image showing an abrupt interface between the UO_2_ film and the LSAT substrate. g) Fourier transforms taken from (e) with crystallographic orientations labeled.

Atomic force microscopy images show that the film surfaces are atomically smooth, presenting steps and terraces, as shown in **Figures** **3**a–d. The root mean square (RMS) surface roughness, measured over the area of 5 × 5 µm^–2^, is found to be 1.5(4), 1.3(5), 1.6(2), and 1.4(2) Å for the films grown on STO, LSAT, LAO, and YAO substrates, respectively. High‐resolution scanning transmission electron microscopy (STEM) was used to characterize the film microstructure and the quality of the film‐substrate interface of the UO_2_ films on LSAT and YAO. Figure 3e shows a cross‐sectional STEM image of the UO_2_/LSAT sample. A relatively sharp interface with 1–2 unit cell intermixing is seen between the substrate and the UO_2_ film. Since the UO_2_/LSAT system has almost no lattice mismatch, it is not surprising that the film/substrate is free of misfit dislocations, due to the nearly homoepitaxial growth. The STEM image in Figure 3f shows the atomic lattice matching between UO_2_ and LSAT near the substrate‐film interface, indicating the high‐quality epitaxial growth. The in‐plane epitaxial relationship from the fast Fourier transform images (Figure 3g) is consistent with XRD results. A STEM image of the UO_2_ film on YAO is also provided in the supporting information (Figure S1, Supporting Information).

**Figure 4:**
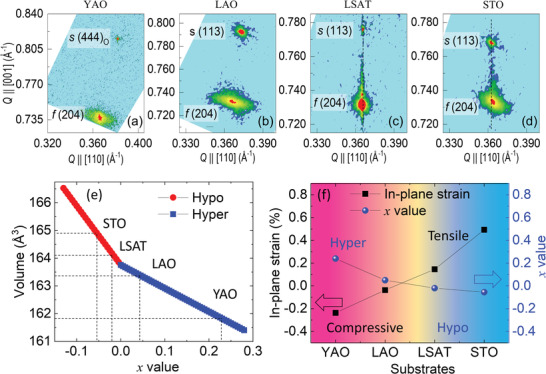
RSMs around (444)_O_ orthorhombic plane of a) YAO and (113) pseudocubic plane of b) LAO, c) LSAT and d) STO substrates. All the substrate and film peaks are shown by ***s*** and f signs, respectively. e) The oxygen stoichiometry deviation (x) dependent volume for UO_2+x_. The data (blue and red line) is adapted from Ref. [39]. f) The correlation between the in‐plane strain and the *x* value for UO_2+x_ films grown on different substrates.

To characterize the strain states, the X‐ray reciprocal space maps (RSMs) are recorded around (113)*c* (where the index *c* refers to the cubic perovskite sublattice) planes of STO, LSAT, and LAO, and (444)*_O_* (where the index *o* refers to the orthorhombic perovskite sublattice) plane of YAO, as shown in **Figures** **4**a–d. RSMs were recorded on the same samples characterized in Figure 2a. RSMs show (204) peak from the UO_2_ films in the lower region and substrate peaks in the upper region for all samples. The out‐of‐plane lattice parameter (*a*_⊥_), in‐plane lattice parameter (*a*_∥_), lattice mismatch, strain, and Poisson ratio of these films are calculated and shown in Table 1.^[^ ^27^ ^]^ The films grown on STO and LSAT substrates are almost fully strained on the substrate as the horizontal peak positions of the UO_2_ films almost coincide with those of the substrates. The in‐plane lattice parameter of UO_2_ films on STO is 5.498 Å with an in‐plane tensile strain of 0.49% and the out‐of‐plane compressive strain of ‐0.29%. The Poisson ratio of UO_2_ films on STO is calculated to be ≈0.23, which is slightly lower than that in polycrystalline UO_2_ bulk (≈0.33).^[^ ^38^ ^]^ The reasonable Poisson ratio indicates that the strain engineering of UO_2_ on STO substrate is valid. For films grown on both LAO and YAO substrates (both large in‐plane compressive misfit strain), the shifts of the films’ peaks to lower *Q*∥[110] values suggest significant relaxation of the in‐plane lattice parameter of the film at the given thicknesses. The UO_2_ films are still in‐plane compressively strained to YAO and LAO substrates with a lattice parameter of 5.458 and 5.469 Å, respectively. And the corresponding in‐plane compressive strain is ‐0.24% and ‐0.04%, respectively. Interestingly, RSM results show the out‐of‐plane lattice of UO_2_ in these two cases are smaller than UO_2_ bulk, contradicting the Poisson theory. Oxygen vacancy formation is a common strain relaxation mechanism in oxides. Different from most oxides, UO_2+x_ can show both hyperstoichiometry (x>0) and hypostoichiometry (x<0), which directly correlate to the lattice parameter.^[^ ^39^ ^]^ A reasonable explanation for both *a*_⊥_ and *a*_∥_ of uranium oxide films being smaller is that the in‐plane compressive strain makes the film become hyperstoichiometric and the unit cell will be shrunk in this hyperstoichiometric regime. A general correlation between unit cell volume and oxygen stoichiometry deviation (*x*) are shown in Figure 4e. In the hyperstoichiometric regime (*x*>0), the lattice parameter (volume) is less than the bulk value, while in the hypostoichiometric regime (*x*<0), the lattice parameter (volume) is larger than the bulk value. Applying the obtained volume on our thin films in Figure 4e, the *x* value can be estimated. We conclude that UO_2+_ *_x_* films on STO and LSAT are hypostoichiometric with *x*<0 while UO_2+_ *_x_* films on LAO and YAO are hyperstoichiometric with *x*>0. In other words, in‐plane tensile strain makes UO_2_ films hypostoichiometric with loss of oxygen (*x*<0) while in‐plane compressive strain makes UO_2_ hyperstoichiometric with the incorporation of excess oxygen (*x*>0) in the lattice. Substrate‐dependent *x* value and in‐plane strain are shown in Figure 4f. It is shown that the *x* value is inversely proportional to the in‐plane strain. Therefore, in UO_2_ thin films, the strain is accommodated by oxygen content incorporation. It should be noted that since LSAT provides almost zero strain, the hypostoichiometric condition of UO_1.98_/LSAT is dominated by the slightly oxygen‐poor growth condition. The hypostoichiometric condition of UO_1.95_/STO will be dominated by the in‐plane tensile strain. The hyperstoichiometric conditions of UO_2.05_/LAO and UO_2.23_/YAO are dominated by the in‐plane compressive strain.

**Figure 5:**
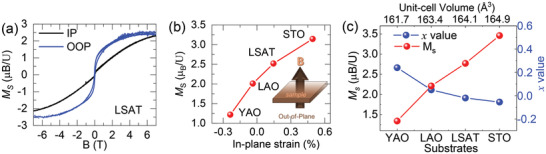
a) Comparison of out‐of‐plane and in‐plane *M(H)* for the UO_2_ film on LSAT measured at 10 K. The magnetic easy axis is oriented along the OOP direction in compressive‐strained UO_2_ film on STO. b) In‐plane strain‐dependent OOP saturated magnetization (*M_S_*) for UO_2+x_ films grown on YAO, LAO, LSAT, and STO substrates measured at 10 K. Inset shows the OOP *M(H)* measurement configuration. c) The growth substrate and unit‐cell volume of UO_2+x_ films dependent *M_S_* and *x* value.

To investigate the effect of substrate on the physical properties, the electric transport and magnetic properties of UO_2_ films are investigated. All films show insulating behavior (resistivity inversely proportional to temperature), as confirmed by temperature‐dependent resistivity measurements [*ρ*(*T*)] (Figure S2, Supporting Information). To examine the effect of the strain and unit cell volume on the magnetic properties, the magnetization (*M*) versus magnetic field (*H*) is performed by applying the field along the out‐of‐plane and in‐plane directions to the samples. **Figures** **5**a shows a representative out‐of‐plane and in‐plane *M*(*H*) for the UO_1.98_ film on LSAT taken at 10 K. The magnetic easy axis is found along the out‐of‐plane direction by comparing the in‐plane and out‐of‐plane *M*(*H*) loops. The UO_1.98_ film on the LSAT substrate shows a saturated magnetization (*M_S_*) of ≈2.5 µ_B_/U. All *M*(*H*) curves are shown in Figure S3 (Supporting Information) It will be interesting to discuss the origin of the ferromagnetic ground state in UO_2+_ *_x_* thin films as the UO_2_ bulk has an antiferromagnetic ground state. It was reported that pressure can induce weak ferromagnetism in UO_2_ bulk.^[^ ^40^ ^]^ We speculate both epitaxial biaxial strain and strain relaxation‐induced point defects could be the main factors for the induced ferromagnetism in UO_2+_ *_x_* thin films. For the UO_1.98_ film on LSAT substrate with limited epitaxial strain, point defects could be the dominating factor. Figure 5b,c shows *M_S_* can be correlated with the in‐plane strain and *x* value. *M_S_* increases with increasing the in‐plane tensile strain or with increasing the unit cell volume. Different from hydrostatic pressure applied to bulk, epitaxial biaxial strain induces tetragonality and affects Jahn‐Teller distortion. Such biaxial strain has been widely correlated with magnetism modulation in perovskite oxides.^[^ ^41^ ^]^ In addition, *M_S_* increases with decreasing the *x* value. In other words, hypostoichiometric UO_2+_ *_x_* with *x*<0 presents higher *M_S_* while hyperstoichiometric UO_2+_ *_x_* with *x*>0 shows smaller *M_S_*. We argue that point defects such as oxygen vacancies and oxygen interstitials could be one of the major factors in inducing magnetism. It was reported that the oxygen deformation can result in the four‐sublattice model and noncollinear spin configuration.^[^ ^22^ ^]^ Strain relaxation‐induced point defects such as oxygen/uranium vacancies and oxygen/uranium interstitials could distort the magnetic structure and result in uncompensated magnetic moments.

**Figure 6:**
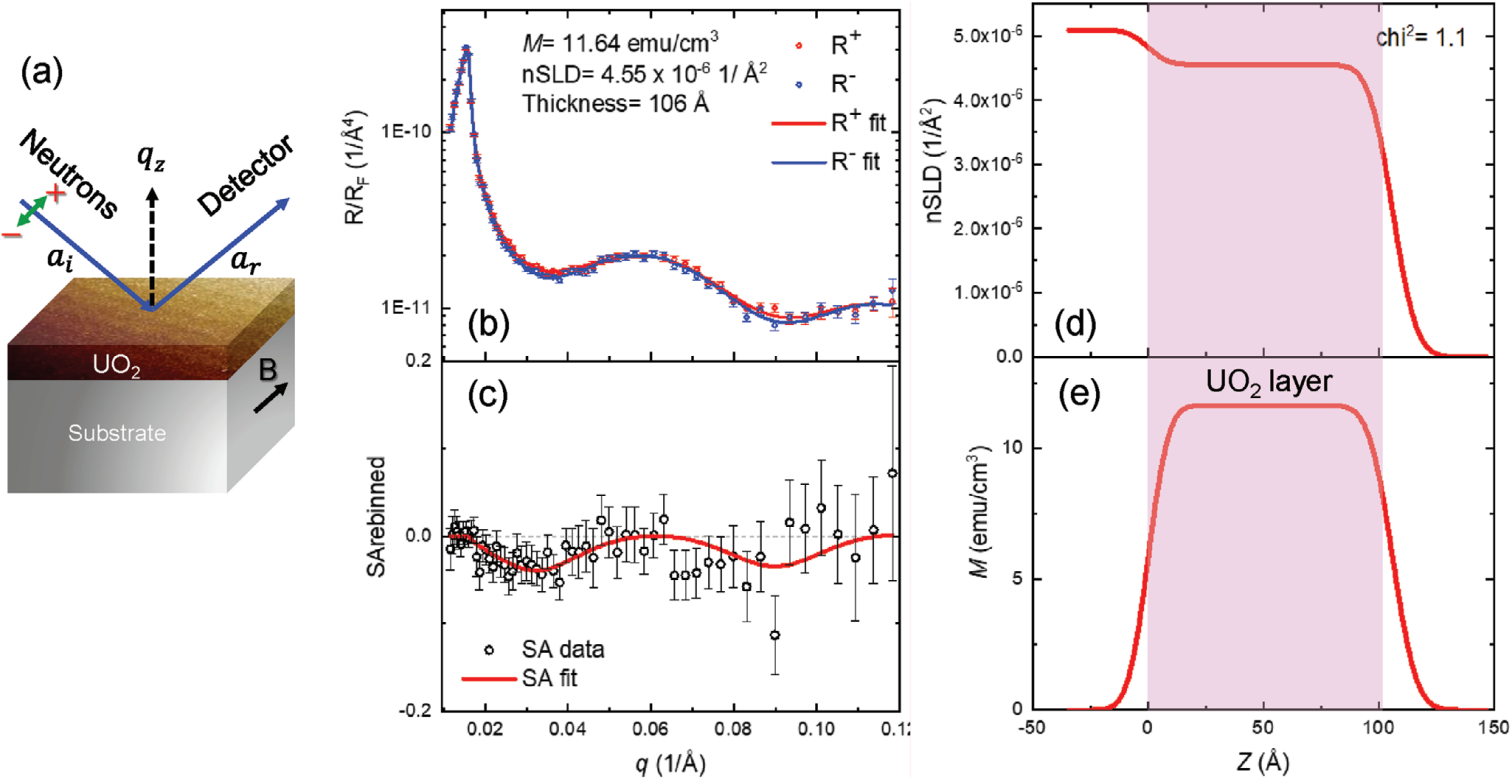
a) A schematic diagram of the experimental setup for PNR measurements. b) Reflectivity curves for spin‐up (R^+^) and spin‐down (R^−^) polarized neutrons as a function of wave vector ***q***_***z***_ (upper panel). Reflectivity curves are normalized to the Fresnel reflectivity, *R*_F_ = 16*π* ^2^/q^4^. c) Spin asymmetry (SA), calculated by (R^+^ − R^−^)/(R^+^ + R^−^), where the error bars represent standard deviation. d) The depth profile for nuclear scattering length density (nSLD). e) The magnetization calculated for a comparison to the bulk magnetometry results.

To confirm the ferromagnetic ground state, the magnetization profile was measured by polarized neutron reflectivity (PNR). As shown in **Figure** **6**a, the wave vector transfer‐dependent reflectivity *R*(*q_z_*) probes the depth profile of the magnetic induction, and the sensitivity to the in‐plane magnetic induction is revealed by differences in the reflectivity curves for neutrons of opposite polarizations with neutron spin parallel (+) and antiparallel (−) to the external magnetic field at the same *q_z_* value. Figure 6b shows reflectivity curves obtained from an 11 nm UO_2_ film on YAO, for both spin directions at *T* = 10 K and a magnetic field of 4.8 T was applied in the film plane. (Note, scattering of the neutron beam requires $\;\vec{q_{z}}$ to be perpendicular to the magnetization, and specular reflectometry requires $\vec{q_{z}}$ to be perpendicular to the film's surface, see Figure 6a). Note that the 11 nm film used in PNR measurements and the 17 nm film presented in Figure 2a have similar strain states and lattice parameters. The interference of the neutrons reflected from both the surface and the UO_2_/YAO interface leads to the fringes visible at higher *q_z_*. A difference between spin up and down is detected as shown in Figure 6c. To analyze the PNR measurements quantitatively, the internal magnetization distribution is modeled using Gaussian profiles. Figure 6d shows excellent agreement between the simulation (red line) and the experimental data. A uniform magnetization depth profile yields reflectivity curves that are consistent with the data (Figure 6e). Notably, there is no need to introduce deviation of the magnetization at or near the surface or buried interface other than that which is consistent with surface and interface roughness. However, the average moment per U of 〈*µ*_*U*_〉 ≈ 12 kA m^–1^ (≈ 0.21 µ_B_ U) from PNR is smaller than the value of ≈ 19 kA m^–1^ (≈ 0.34 µ_B_ U) from *M*(*H*) measurements (Figure S4, Supporting Information). We believe that the ground state ferromagnetism in UO_2+_ *_x_* films probably occurs due to the canting of the magnetic moment, where the point defects and epitaxial strain affects the canting angle and thereby net magnetization of the film. To clarify this point, further measurements aimed at quantifying the angle of spin canting will be needed.

## 3 Conclusion

In summary, strain engineering is explored in high‐quality epitaxial UO_2_ thin films on different substrates. XRD and STEM results demonstrate excellent crystalline quality and smooth interfaces of the strained UO_2_ films. Field‐dependent magnetometry shows a ferromagnetic behavior of UO_2_ thin films. Neutron scattering confirmed uniformly distributed ferromagnetism across the thickness of UO_2_/YAO sample. UO_2+_ *_x_* thin films, strained on STO substrate (in‐plane tensile strain), are hypostoichiometric with *x*<0 while UO_2+_ *_x_* thin films grown on LAO and YAO substrates with in‐plane compressive strain are hyperstoichiometric with *x*>0. The incorporation of point defects is a critical way to accommodate epitaxial strain in UO_2_ thin films. Epitaxial strain and strain relaxation induced point defects such as oxygen/uranium vacancies and oxygen/uranium interstitials could distort the noncollinear magnetic structure and result in magnetic moments in UO_2+_ *_x_* thin films. These results will guide strain engineering of other actinide oxide thin films and understand physical behaviors.

## 4 Experimental Section

### Epitaxial Film Growth

A ceramic stoichiometric target of UO_2_ was fabricated by a conventional ceramic cold pressing and sintering process. The detailed target synthesis conditions were described elsewhere.^[^ ^37^ ^]^ Pulsed laser deposition (PLD) was used to grow UO_2_ thin films on different single crystal perovskite substrates. The base pressure of the PLD chamber was 6 × 10^−7^ Torr. A KrF excimer laser (Lambda Physik LPX 300, *λ* = 248 nm, 1−5 Hz) pulses were focused on a stoichiometric, depleted UO_2_ target at a repetition of 4 Hz and at an energy density of 1.5 J cm^–2^. The target‐substrate distance was set at 5 cm^–1^. The films were grown at a temperature of 580 °C with an argon gas partial pressure of 100 mTorr. After deposition, films were cooled down to room temperature at a rate of 10 °C min^–1^ maintaining the argon gas partial pressure of 100 mTorr.

### Structure, Transport, and Magnetic Measurements

The crystal structure and growth orientation of the films were characterized by X‐ray diffraction (XRD) using a four‐circle high‐resolution X‐ray diffractometer (X'Pert Pro, Panalytical) (Cu K*α*_1_ radiation). Atomic force microscopy (Nanoscope III AFM) was used in the tapping mode to monitor the surface morphology of the as‐grown films. Magnetic hysteresis loops were recorded using a physical property measurement system (PPMS, Quantum Design) with a vibrating sample magnetometer (VSM). The macroscopic electrical transport properties of the films were measured by a four‐probe method using PPMS. Deep ultraviolet Raman spectroscopic measurements were conducted in reflection mode using 244 nm excitation (Cambridge Laser Laboratories LEXEL Quantum 8 SHG continuous wave tunable Argon ion laser), in a Horiba LabRAM HR Evolution microscope, configured with a 2400 gr mm^−1^ grating blazed at 250 nm, 200 µm confocal hole diameter, and a 74 ×, 0.65 N.A. reflective objective. Spectral calibration was performed using the 1332.5 cm^−1^ band of a synthetic Type IIa diamond.^[^ ^42^ ^]^ The microstructure of the films and interface quality between film and substrate were studied by an FEI Titan 80–300 scanning transmission electron microscope (S/TEM) operated at an accelerating voltage of 300 kV. Cross‐sectional specimens oriented along the [100] LSAT direction for HRTEM analysis were prepared using an FEI Helios 600 dual‐beam focused ion beam/scanning electron microscope (FIB/SEM). Samples were lifted out in situ and attached to a half‐grid. All thinning work was performed at 30 kV, a final polishing was conducted at 2 kV in order to remove the amorphous damage layer from the lamella's free surfaces. High‐resolution STEM images were captured with a ≈100 pA beam current and a probe semi‐convergence angle of 10.0 mrad.

### Polarized Neutron Reflectivity Measurements

The PNR measurements were performed at the BL‐4A beamline of the Spallation Neutron Source (SNS) at Oak Ridge National Laboratory. All data were collected at a temperature of 10 K, well below the magnetic transition temperature (*T*_N_) of UO_2_, in a helium exchange gas using a closed‐cycle cryostat. The data were recorded after first cooling the sample in a field of 4.8 T, and then measured in this field with the field applied parallel to the sample plane and to the wave vector of the incident neutron beam. The diffuse neutron background was collected using a 2D position‐sensitive detector. The background around the specular reflectivity was used to estimate the background beneath the specular reflectivity and subtracted from the total intensity. Corrections accounting for the inefficiencies of the neutron beam polarizer and neutron spin flippers were applied to the data yielding the spin‐dependent neutron beam reflectivity *R* ^±^(*q_z_*). Details and code for steps in processing the data are provided in Ref. [31, 43]. Various models were fitted to the data using GenX.^[^ ^44^ ^]^

## Conflict of Interest

The authors declare no conflict of interest.

## Supporting information

Supporting Information

## Data Availability

The data that support the findings of this study are available from the corresponding author upon reasonable request.
