# Supplementary material for: Induced Ferromagnetism in Epitaxial Uranium Dioxide Thin Films
Source: Adv Sci (Weinh). 2022 Oct 9;9(33):2203473. doi: 10.1002/advs.202203473 (PMC9685444; doi:10.1002/advs.202203473)
Supplement: Supplementary file 1 — Supporting Information [file ADVS-9-2203473-s001.pdf]

ADVANCED SCIENCE
Open Access

Supporting Information

for *Adv. Sci.*, DOI 10.1002/advs.202203473

Induced Ferromagnetism in Epitaxial Uranium Dioxide Thin Films

Yogesh Sharma, Binod Paudel, Amanda Huon, Matthew M. Schneider, Pinku Roy, Zachary Corey, Rico Schönemann, Andrew C. Jones, Marcelo Jaime, Dmitry A. Yarotski, Timothy Charlton, Michael R. Fitzsimmons, Quanxi Jia, Michael T. Pettes, Ping Yang and Aiping Chen**

Supporting Information

Induced Ferromagnetism in Epitaxial Uranium Dioxide Thin Films

Yogesh Sharma,[1,2,*] Binod Paudel,[1] Amanda Huon,[3,4,#] Matthew M. Schneider,[5] Pinku Roy,[6] Zachary Corey,[6] Rico Schöenemann,[7] Andrew C. Jones,[1] Marcelo Jaime,[7] Dmitry A. Yarotski,[1] Timothy Charlton,[3,4] Michael R. Fitzsimmons,[3,4,8] Quanxi Jia,[6] Michael T. Pettes,[1] Ping Yang,[2] and Aiping Chen[1,*]

[1] Center for Integrated Nanotechnologies (CINT), Los Alamos National Laboratory, Los Alamos, NM 87545, USA

[2] Glenn T. Seaborg Institute, Los Alamos National Laboratory, Los Alamos, NM 87545, USA

[3] Neutron Scattering Division, Oak Ridge National Laboratory, Oak Ridge, TN 37831, USA

[4] Materials Science and Technology Division, Oak Ridge National Laboratory, Oak Ridge, TN 37831, USA

[5] Materials Science and Technology Division, Los Alamos National Laboratory, Los Alamos, NM 87545, USA

[6] Department of Materials Design and Innovation, University at Buffalo, The State University of New York, NY,14260, USA

[7] National High Magnetic Field Laboratory (NHMFL), Los Alamos National Laboratory, Los Alamos, NM 87545, USA

[8] University of Tennessee, Department of Physics and Astronomy, Knoxville TN 37996, USA

* E-mail: yks181086@gmail.com; apchen@lanl.gov

# Present address: Department of Mathematics, Physics, and Statistics, University of the Sciences in Philadelphia, PA, 19104, USA

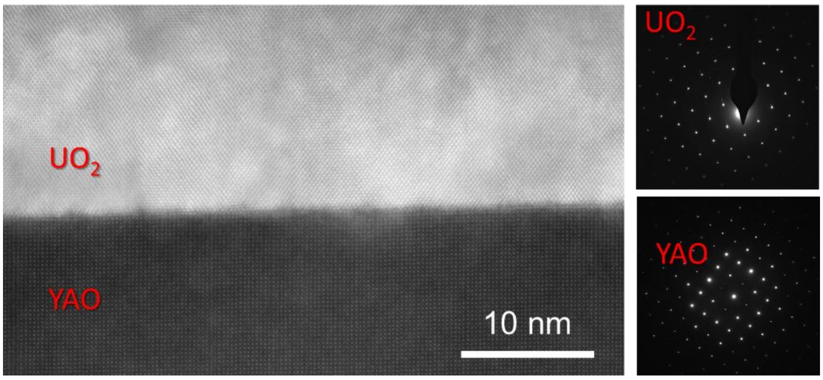

Figure S1. Epitaxial (001) UO_2 films deposited on a single crystal $(110)_O$ YAO substrate. Left) a cross sectional STEM image. Right) the selected-area electron diffraction patterns for UO_2 and YAO substrates.

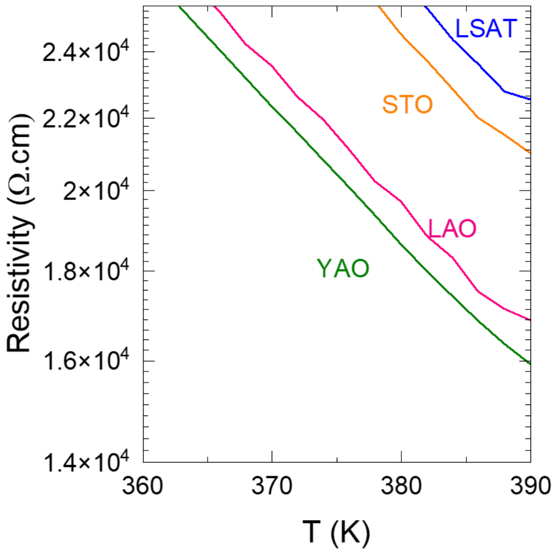

Figure S2. Resistivity (ρ) as a function of temperature (T) for the UO_2 thin films on different substrates.

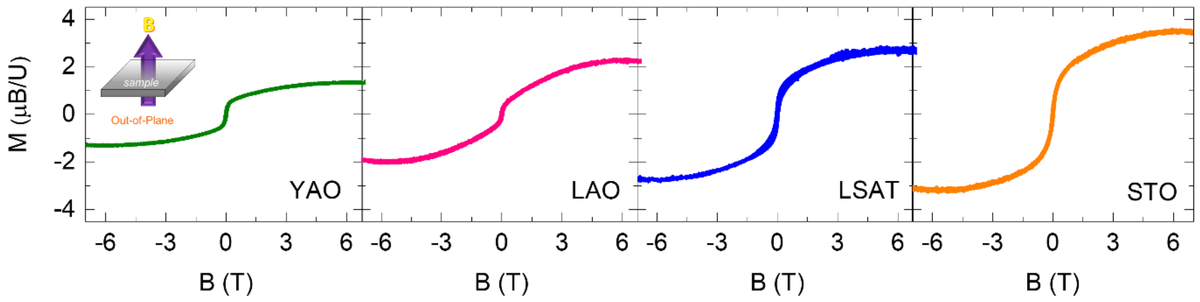

Figure S3. Out-of-plane magnetization as a function of applied magnetic field (*M*-*H*) for UO_2 films grown on YAO, LAO, LSAT, and STO substrates.

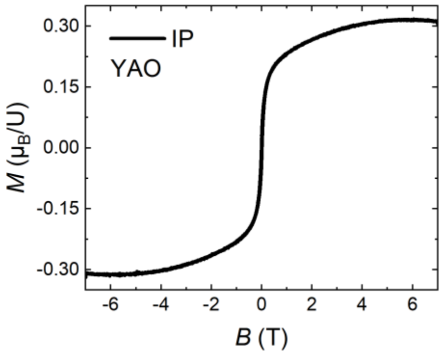

Figure S4. In-plane magnetization as a function of applied magnetic field (*M*-*H*) for UO_2 films grown on YAO.
